# The Interplay between Cardiovascular Risk, Cardiovascular Events, and Disease Activity in Primary Sjögren’s Syndrome: Is Uric Acid the Missing Link?

**DOI:** 10.3390/nu15071563

**Published:** 2023-03-23

**Authors:** Alessia Alunno, Francesco Carubbi, Francesco Maria Mariani, Cecilia Martini, Elena Campanozzi, Claudio Ferri

**Affiliations:** Internal Medicine and Nephrology Division, Department of Life, Health & Environmental Sciences, San Salvatore Hospital, University of L’Aquila, ASL 1 Avezzano-Sulmona-L’Aquila, 67100 L’Aquila, Italy

**Keywords:** cardiovascular risk, cerebrovascular disease, Sjögren’s syndrome

## Abstract

(1) Background: Uric acid is a well-known cardiovascular (CV) risk factor in the general population but its role in the setting of rheumatic diseases other than gout is unclear. (2) Methods: This is a retrospective study investigating a cohort of 105 pSS patients recording clinical, serological, and CV-related variables including adherence to the Mediterranean diet. (3) Results: We observed a strong relationship between disease activity, interstitial lung disease (ILD), and CV events. The association between ILD and CV events was dependent on higher SUA levels but independent of other traditional CV risk factors. All three cases of previous non-fatal stroke were reported by females aged <65 years, with higher SUA levels, and two of them also had pSS-ILD. Forty (38%) patients had a 10-year risk of fatal and non-fatal CV disease events beyond the cut-off recommended for their age, and using the correction factor of 1.5 currently applied only to rheumatoid arthritis, we could better identify patient subsets characterized by different CV risk profiles including different SUA levels. (4) Conclusions: This study is the first to investigate in depth the role of SUA in the CV scenario of pSS. Our findings underpin the importance of assessing SUA levels in pSS in addition to the other traditional CV risk factors and to consider applying the correction factor for CV risk assessment tools to achieve a better stratification of CV risk.

## 1. Introduction

An unhealthy diet, with or without impaired renal urate excretion, is the most frequent cause of hyperuricemia [1]. Despite its pivotal role in the pathogenesis of gout, the clinical relevance of serum uric acid (SUA) levels goes well beyond the simple association with gout and/or nephrolithiasis [2,3]. Previous studies have pointed to hyperuricemia as a cardiovascular (CV) risk factor in the general population; therefore, the 2018 guidelines of the European Society of Cardiology (ESC) and the European Society of Hypertension (ESH) included the assessment of uricemia among the screening tests to be performed in hypertensive patients [4]. Furthermore, in view of the association between hyperuricemia and mortality (both CV and all-cause) and CV events, it is conceivable that CV damage begins with levels of uricemia lower than 6 mg/dL. In this context, the first results of the URRAH (Uric Acid Right for Heart Health) study identified an SUA threshold value of 4.7 mg/dL for all-cause mortality and 5.6 mg/dL for CV mortality [5]. In addition, in the same study, SUA was an independent risk factor for cerebrovascular (CBV) events after adjusting for potential confounding variables, including arterial hypertension (AH), and it identified a valid prognostic cut-off value (>4.79 mg/dL). Concordantly, SUA levels >5.34 mg/dL (confidence interval, CI 4.37–5.6, sensitivity 52.32, specificity 63.96, *p* < 0.0001) were the univariate prognostic cut-off value for all heart failure, whereas SUA levels >4.89 mg/dL (CI 4.78–5.78, sensitivity 68.29, specificity 49.11, *p* < 0.0001) were the univariate prognostic cut-off value for fatal heart failure [6].

Primary Sjögren’s syndrome (pSS) is a chronic inflammatory disease with a 9:1 female-to-male ratio and a prevalence ranging between 0.5 and 1% in the general population. Despite showing a tropism for exocrine glands, pSS can virtually involve any organ and system and cause extra-glandular clinical manifestations in up to 75% of patients [7,8,9]. Therefore, although the clinical picture is dominated by signs and symptoms of mucosal dryness, extra-glandular involvement may lead to a more severe disease course with additional manifestations such as arthritis, neuropathies, and purpura to cite a few. B-cell lymphoma is the most severe complication of pSS occurring in about 5% of patients.

In addition, pSS is burdened by higher CV morbidity and mortality compared to the general population like other chronic inflammatory diseases [10]. Previous studies have demonstrated that patients with pSS display traditional CV risk factors such as hypercholesterolemia, AH, and obesity [11]. However, the excess CV risk they have is in part attributable to underlying chronic inflammatory diseases as well the possible effect of drugs such as glucocorticoids.

The only available study that has explored SUA levels in pSS is a retrospective analysis showing that SUA levels may be associated with the onset of AH in a Chinese population [12]. However, they did not look into the role of SUA on overall CV risk or the development of CV events, nor did they investigate any relationship with disease activity.

Based on the existing data about SUA in the general population, we postulated that SUA may be of particular relevance in the pSS scenario. Therefore, since data on pSS in this area are lacking, we aimed to explore how SUA levels correlate with other CV risk factors, previous CV events, and disease-related features in patients with pSS and without gout and/or nephrolithiasis.

## 2. Materials and Methods

### 2.1. Population Study and Inclusion Criteria

A cross-sectional study was conducted recruiting consecutive patients with pSS according to the 2016 American College of Rheumatology/European League Against Rheumatism (ACR/EULAR) classification criteria of aged 18 years or above [13]. The exclusion criteria were gout and/or nephrolithiasis, ongoing treatment with urate-lowering agents, one or more systemic/organ-specific autoimmune diseases other than pSS, and a current or recent history of neoplasm. The sample size calculation estimated that approximately 130 subjects had to be enrolled to achieve a power of 80% with alpha (probability of type I error) = 0.05. This value was obtained using reference data on the prevalence of CV risk factors and CV diseases in the general population and in the population of patients with various rheumatological diseases. Due to the above-mentioned exclusion criteria, the number of eligible patients we enrolled was slightly lower than the target estimated by the sample size calculation. The study was approved by the local Institutional Review Board.

### 2.2. Clinical and Serological Features

#### 2.2.1. Demographic and Disease-Specific Features

Disease activity was calculated with the EULAR Sjögren’s syndrome disease activity index (ESSDAI) [14] while the patient perception of symptoms (overall dryness, ocular dryness, oral dryness, pain, and fatigue) was reported on 0–10 visual analog scales (VAS). The overall dryness, pain, and fatigue VAS were also used to calculate the EULAR Sjogren’s Syndrome Patient Reported Index (ESSPRI) [15]. Anthropometric parameters recorded were height and weight and used to calculate the body mass index (BMI) [weight (kg) divided by height squared (m^2^), kg/m^2^].

#### 2.2.2. Cardiovascular Risk Factors

The following CV disease risk factors were considered: smoking (defined as previous/current/no use of at least one cigarette/day), AH (physician diagnosis and/or prior/ongoing antihypertensive therapy), hypercholesterolemia (total serum cholesterol level >200 mg/dL in at least three assays and/or prior/ongoing lipid-lowering therapy), hypertriglyceridemia (serum triglyceride level >150 mg/dL in at least three assays and/or prior/ongoing lipid-lowering therapy), high-density lipoprotein cholesterol (HDL-c) level (reduced <40 mg/dL, normal 40–60 mg/dL, increased >60 mg/dL in at least three assays), low-density lipoprotein cholesterol (LDL-c) level (increased >115 mg/dL in at least three assays), type 2 diabetes mellitus (DM) (ongoing treatment with insulin or oral hypoglycemic agents and/or glucose level >126 mg/dL in at least two fasting glycaemia tests), and obesity (according to BMI). The degree of obesity was established as follows: 25–30 kg/m^2^ (overweight), 30–34.9 kg/m^2^ (grade I obesity), 35–39.9 kg/m^2^ (grade II obesity), and ≥40 kg/m^2^ (grade III obesity or severe obesity). Metabolic syndrome was defined according to the National Cholesterol Education Program (NCEP) Adult Treatment Panel III (ATP III) criteria [16]. SUA was measured upon recruitment. In eligible patients, the 10-year risk of fatal and non-fatal CV disease events was calculated using the HeartScore^®^ online tool of the European Society of Preventive Cardiology that incorporates the SCORE2 and SCORE2-OP risk prediction algorithms [17]. These algorithms estimate 10-year fatal and non-fatal CV disease risk in individuals in Europe without previous CV events or diabetes aged 40–69 years (SCORE2) and aged over 70 years (SCORE2-OP). The HeartScore^®^ online tool is also calibrated to four European risk regions, based on age- and sex-standardized CV disease mortality rates.

#### 2.2.3. Cardiovascular Events

CV events, namely myocardial infarction, angina, heart failure, cerebrovascular events, and arteriosclerotic vascular disease were recorded. CV events were recorded only if the diagnosis was confirmed by hospital discharge records and/or available specific laboratory and diagnostic examinations.

#### 2.2.4. Adherence to the Mediterranean Diet

Adherence to the Mediterranean Diet over the previous 12 months was assessed with the 14-item PREvencion con DIeta MEDiterranea’ (PREDIMED) tool [18]. A total score ≤5 indicates poor adherence, a score between 6 and 9 indicates medium adherence, and a score ≥10 indicates good adherence. The 28-item Mediterranean Lifestyle (MEDLIFE) Index was also used [19]. The MEDLIFE is structured as follows: block 1 includes 15 questions on Mediterranean food consumption (e.g., How many servings of fish or seafood portions do you consume per week?); block 2 includes 7 questions on Mediterranean dietary habits (e.g., Do you usually choose whole grain products?); and block 3 includes 6 questions on physical activity, rest, social habits, and conviviality (e.g., Do you engage in physical activity (>150 min/week or 30 min/day)?).

### 2.3. Statistical Analysis

The data were analyzed with IBM SPSS v.28.0. The normality of the data was assessed with the Kolmogorov–Smirnov test. As detailed throughout, the χ^2^ test, Mann-Whitney U test, or Kruskal-Wallis test with Dunn’s test for multiple comparison post hoc was used to compare the groups. Univariate regression and multivariate binary logistic regression were employed to identify any association between the variables. Bivariate correlation (Spearman’s ρ) was also performed. The significance level was two sided and set at *p*-values less than 0.05.

## 3. Results

### 3.1. Analysis of the Full Cohort

One hundred and five patients with pSS (100 females and 5 males) were eligible for enrollment. Demographic and clinical and laboratory features, including CV risk factors and CV events, are shown in Table 1. The mean (standard deviation, SD) value of SUA was 4.4 ± 1.1 in the whole cohort. According to the prognostic cut-off value for CBV diseases of the URRAH study, 24 (23%) patients had SUA levels ≥4.79 mg/dL. In addition, seven (7%) patients had SUA levels ≥5.6 mg/dL, the cut-off value for CV mortality identified by the URRAH study [5].

As shown in Table 2, patients with SUA levels ≥4.79 mg/dL had a significantly higher BMI (27.2 vs. 24.6, *p* = 0.009), higher triglycerides levels (134.7 vs. 98.9, *p* = 0.009), a higher number of CV risk factors (>2 risk factors in 42 vs. 20% of patients), and were more frequently current or former smokers (51 vs. 22%) compared to patients with SUA levels < 4.79 mg/dL.

With regard to previous CV events, all cases of stroke occurred in patients with SUA levels ≥4.79 mg/dL while no difference was observed regarding the other types of events or the total number of events. The prevalence of stroke in our population (2.8%, age range 26–86 years) was higher than that of the Italian general population publicly available on the Progetto Cuore website (0.8% females-1% males, age range 35–79 years, period 2008–2012) [20]. Of interest, all the three patients who had experienced a stroke were aged 65 or below at study recruitment.

In addition, patients with SUA levels ≥4.79 mg/dL had higher disease activity compared to patients with SUA levels <4.79 mg/dL (mean ESSDAI value 9 vs. 5.5, *p* = 0.005), and when assessing the individual ESSDAI domains, glandular, pulmonary, and hematological manifestations were more frequently observed in patients with SUA levels ≥4.79 mg/dL (Supplementary Table S1) compared to patients with SUA levels <4.79 mg/dL.

We then sought to investigate the features of the seven patients with SUA levels ≥5.6 mg/dL, the cut-off value for CV mortality identified by the URRAH study. As shown in Supplementary Table S2, when comparing patients with SUA levels <4.79 mg/dL, 4.79 mg/dL ≤ SUA < 5.6 mg/dL or ≥5.6 mg/dL, we observed that increasing SUA levels paralleled an increase in disease activity (mean ESSDAI value 5.5 vs. 8.7 vs. 9.9, *p* = 0.019) while patient-reported outcomes were comparable across the three groups. When assessing the ESSDAI domains, a progressively higher prevalence of glandular (13 vs. 23 vs. 57%, 0.01) and pulmonary (0 vs. 18 vs. 28%, *p* < 0.001) manifestations was observed in groups with higher SUA levels (Supplementary Table S3).

With regard to the Mediterranean diet, the majority of patients had a medium or good adherence (*N* = 98/105; 93%); however, no differences in the PREDIMED or any of the MEDLIFE scores were observed across groups with different SUA levels.

### 3.2. Analysis of pSS Patients with Arterial Hypertension

As mentioned above, SUA has been included by ESC/ESH among the screening tests to be performed in hypertensive patients [4]; therefore, we analyzed in more detail pSS hypertensive patients (Table 3).

The prevalence of AH in our pSS population was 44% and when compared to non-hypertensive patients, the 46 pSS patients with AH resulted older and with a longer disease duration. They had a higher BMI, albeit with a comparable prevalence of obesity, a worse lipid profile with higher total cholesterol and LDL-c, higher triglycerides and lower HDL-c. They were more frequently current or former smokers, had a higher number of CV risk factors in addition to AH, and where more likely to have a history of previous CV events. Of the three patients with a history of stroke, only two were hypertensive.

When evaluating the dietary habits, pSS patients with AH had significantly lower mean (SD) levels of MEDLIFE total scores compared to non-hypertensive patients (14.8 (3.3) vs. 16.4 (2.9) *p* = 0.02) and this was due to significantly lower levels of block 3 which explores healthy lifestyle habits (2.05 (1.1) vs. 2.9 (1.2) *p* = 0.003). Regarding individual domains of block 3, it emerged that hypertensive patients had more sedentary habits and did not engage in at least 150 min/week or 30 min/day of physical activity. Conversely, blocks 1 and 2 of the MEDLIFE tool and the total PREDIMED score, exploring Mediterranean food and dietary habits, were comparable in hypertensive and non-hypertensive patients. However, a more detailed analysis of individual nutrient intake confirmed the association between AH and lower fish consumption which has been described in the general population [21,22] and which we observed in an independent pSS cohort [23]. In particular, only 15/46 hypertensive patients (32%) consumed at least two servings of fish/seafood per week compared to 35/59 (59%) non-hypertensive patients (*p* = 0.006). Binary logistic regression identified an odds ratio of 2.4 (95% confidence interval 1.1–5.5, *p* = 0.04) of having AH in those with lower fish intake.

When exploring the differences among hypertensive patients based on SUA levels (Table 4), we noticed that hypertensive patients with SUA levels ≥ 4.79 mg/dL were more likely obese (43% vs. 9%, *p* = 0.015), current or former smokers, and with a higher disease activity (mean ESSDAI value 10.1 vs. 5.5 *p* = 0.02). As in the analysis of the full cohort, also within hypertensive patients were glandular, pulmonary, and hematological ESSDAI domains more frequently involved in patients with SUA levels ≥ 4.79 mg/dL.

### 3.3. Analysis of pSS Patients without Previous CV Events

Finally, we focused on pSS patients fulfilling the criteria for the application of the SCORE2 and SCORE2-OP risk prediction algorithms (individuals in Europe without previous CV events or diabetes aged 40–69 years (SCORE2) and aged over 70 years (SCORE2-OP)) (Table 5).

**Figure 1:**
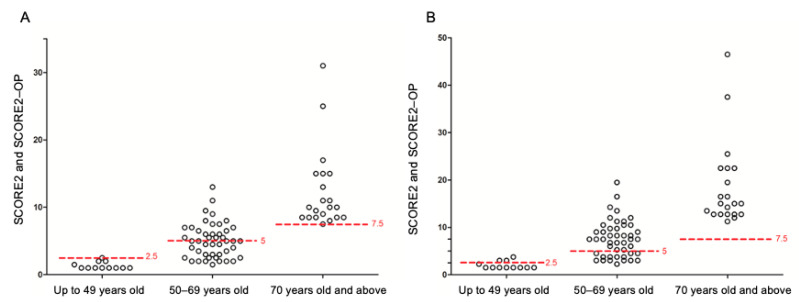
10-year risk of fatal and non-fatal CV disease events calculated using the SCORE2 and SCORE2-OP risk prediction algorithms in the 79 eligible patients with primary Sjögren’s syndrome. Panel (**A**) shows the normal calculation as in the general population; panel (**B**) shows the values using a 1.5 correction factor as currently applied in rheumatoid arthritis. Dotted lines and adjacent numbers show the corresponding cut-off value by age.

By using the online HeartScore^®^ tool, we assessed 79 patients whose characteristics are shown in Table 5. Since we identified that patients eligible for SCORE have lower values of ESSPRI, we analyzed the possible correlation of this variable and CV risk observing a direct correlation between the ESSPRI score and the number of CV risk factors (Spearman’s rho = 0.22, *p* = 0.026). A total of 40 (38%) patients had an estimated risk above the cut-off level recommended by existing guidelines for individuals of the same age, while 6 (7%) had the same value as the cut-off level (Figure 1A). When focusing on SUA levels, we observed that among the patients with a risk below the cut-off level recommended by the guidelines (*N* = 33), six of them (18%) had SUA values ≥4.79 mg/dL, and one of these six patients reached values ≥5.6 mg/dL.

The 2021 update of the ESC guidelines for CV prevention, reinforced the importance that in patients with chronic inflammatory conditions such as rheumatoid arthritis (RA), multiplication of calculated total CV disease risk by a factor of 1.5 should be considered. Although evidence to use the 1.5 correction factor in connective tissue diseases is not yet solid enough to inform recommendations, the experts commented that it seems prudent to take into account the presence of such conditions when there is doubt regarding initiation of preventive interventions since the cumulative disease burden and recent degree of inflammation are important determinants of the risk-enhancing effect [24,25]. Therefore, for speculation purposes, we applied it to the risk scores of our cohort and observed that 10 out of 33 patients were reclassified above the cut-off level recommended by the guidelines for their age (Figure 1B).

We therefore compared the populations whose SCORE remained either within (*N* = 23) or beyond (*N* = 46) the cut-off regardless of the correction factor and the population was reclassified thanks to the correction factor. It emerged that the patients whose SCORE remained within the cut-off regardless of the correction factor are the youngest and show the lowest levels of total cholesterol, LDL-c, and triglycerides as well as the highest HDL-c values (all *p* < 0.05). In addition, only 10/23 (43%) patients had at least one CV risk factor (vs. 6/10 (60%) and 35/46 (76%) in the other populations, *p* = 0.027). None of the 23 patients whose SCORE remained within the cut-off regardless of the correction factor showed more than 2 CV risk factors (vs. 5/10 (50%) and 35/46 (76%) in the other populations, *p* = 0.04). As mentioned above, among the patients with a risk below the cut-off level recommended by the guidelines (*N* = 33), six of them (18%) had SUA values ≥4.79 mg/dL, and one of these six patients reached values ≥5.6 mg/dL. Of interest, these six patients were reclassified above the cut-off level following the application of the 1.5 correction factor.

### 3.4. Domains of Disease Activity, SUA Levels and CV Events

The analysis that we performed in the full cohort and in the different subgroups, showed that disease activity as well as the prevalence of involvement of three ESSDAI domains, glandular, pulmonary, and to a certain extent hematological, paralleled the levels of SUA. Therefore, we explored in more detail the relationship between disease activity, disease domains, SUA, and CV risk/CV events. Binary logistic regression demonstrated that pulmonary domain, but not glandular and hematological domains, is strongly associated with the outcome ‘previous CV event’ (univariate analysis: odds ratio (OR) = 15.1, 95% CI = 1.6–141.8, *p* = 0.018). Although the association between SUA levels ≥ 4.79 mg/dL and the outcome ‘previous CV event’ does not reach statistical significance at univariate analysis, when adding ‘SUA levels ≥ 4.79 mg/dL’ as a covariate, the association between the ESSDAI pulmonary domain and the outcome ‘previous CV event’ was no longer significant. In addition, the higher the number of CV events, the higher the odds of also having pulmonary manifestations (OR = 3.9, 95% CI = 1.5–10.0, *p* = 0.004).

Since in our cohort other variables resulted in being significantly associated with the outcome ‘previous CV event’ at univariate analysis, such as hypercholesterolemia (OR = 10.5 *p* = 0.001) and obesity (OR = 3.7, *p* = 0.018), we included them as covariates in the analysis for the association of the ESSDAI pulmonary domain and the outcome ‘previous CV event’. It emerged that both the ESSDAI pulmonary domain (OR = 18.5, *p* = 0.02), hypercholesterolemia (OR = 5.1, *p* = 0.005), and obesity (OR = 4.2, *p* = 0.02) are independently associated with the occurrence of CV events but the highest OR (namely, strongest association) was that with the ESSDAI pulmonary domain.

Finally, since two out of three patients who developed a stroke had pulmonary manifestations, we computed a logistic regression analysis, albeit recognizing the limitation of the analysis due to the small number of cases, and observed an association between ILD and stroke (OR = 66; *p* = 0.02).

Based on the data about the relationship between ESSPRI and the number of CV risk factors, we sought to investigate whether this was also related to CV events, and in fact, this score is also directly correlated with the number of CV events (Spearman’s rho = 0.24, *p* = 0.015).

## 4. Discussion

Over the last decade, observational studies have provided solid evidence of the association between high SUA levels and high CV risk in the general population [25]. This encompasses both clinical and subclinical CV manifestations and points to SUA as a risk factor worth assessing in the setting of CV prevention [4]. The only available study that has explored SUA levels in pSS is a retrospective analysis showing that SUA levels may be associated with the onset of AH in a Chinese population [12]. However, they did not look into the role of SUA on overall CV risk or the development of CV events, nor did they investigate any relationship with disease activity. We investigated in depth the role of SUA in the scenario of pSS and demonstrated a relationship between SUA levels, CV risk/diseases, and disease activity in pSS.

In line with existing evidence, the pSS patients in our cohort display traditional CV risk factors such as hypercholesterolemia, AH, and obesity [11]. Therefore, it is not surprising that in our cohort 24% of the subjects had already experienced a non-fatal CV event at the time of recruitment in the study. When classifying patients according to SUA levels, we could identify specific subgroups characterized not only by a different metabolic profile but also by different disease activity and disease features.

In our cohort, patients with SUA levels ≥ 4.79 mg/dL displayed higher disease activity than those with lower SUA levels and since both ESSDAI and ClinESSDAI were higher, this suggests that clinical rather than laboratory features (as ClinESSDAI excludes the biological domain) may be more relevant in this setting [26]. In more detail, patients with higher SUA levels have a higher prevalence of pulmonary manifestations, namely interstitial lung disease (ILD) and a relationship between pSS-ILD and the occurrence of previous CV events has also emerged. In line with our findings, a recent study demonstrated that patients with ILD associated with RA display higher levels of SUA and also higher levels of bronchoalveolar lavage fluid (BALF)-UA [27]. A study using an experimental model of ILD demonstrated that bleomycin-induced lung injury triggers local production of UA, thereby activating the NALP3 inflammasome in the lung and perpetuating lung damage [28]. In addition, higher SUA and BALF-UA levels have been associated with worse pulmonary function at spirometry not only in RA-ILD but also in healthy subjects [27,29].

These data may suggest that similar events as described in RA occur in pSS thereby explaining our findings on the relationship between SUA and pSS-ILD.

In fact, the relationship between pSS-ILD CV events was dependent on having SUA levels ≥ 4.79 mg/dL but independent of other traditional CV risk factors such as hypercholesterolemia and obesity at multivariate analysis. Since there is evidence that damaged lungs may be a source of UA [28] and based on the dietary habit questionnaires, we could rule-out excessive intake of purine-rich food in our cohort and it is reasonable to speculate that in patients with pSS-ILD, at least part of circulating SUA may derive from the inflamed lungs and therefore contribute to increased CV risk on top of other risk factors.

When focusing on the type of CV event, we reported a higher prevalence of stroke in pSS compared to the general population. This is in line with a previous Italian study, demonstrating a higher prevalence of stoke in pSS [30]. However, a nationwide study demonstrated that Taiwanese patients with pSS had the same prevalence of stroke compared to the general population. Since the increased CV risk we observe in pSS results from the interplay of traditional and disease-specific risk factors, we believe that different ancestry, different dietary and voluptuary habits, as well as different environmental factors may account for this discrepancy [31]. In our cohort, all three cases of previous non-fatal stroke were reported by female patients with SUA levels ≥ 4.79 mg/dL, and two of them also had pSS-ILD.

A recent meta-analysis showed that SUA is a risk factor for stroke development particularly in healthy females [32] and this fits with our findings. However, the molecular mechanisms underlying this association in the general population are still unclear. To the best of our knowledge, there are no data about an association between ILD and stroke.

With regard to the effect of diet on SUA levels, a recent systematic review highlighted that low-calorie, purine-low, and Mediterranean-style diets induce a low, albeit significant reduction in SUA levels in patients with hyperuricemia/gout. Conversely, studies investigating the effect of CV risk factors were limited and inconclusive [33]. Since in our cohort the proportion of hyperuricemic (>6 mg/dL) patients was <5%, this could explain the fact that no effect on SUA levels was observed according to the degree of adherence to the Mediterranean diet.

Finally, when focusing on pSS patients without previous CV events, we realized that 38% of them had values beyond the cut-off recommended for their age, and if using the correction factor of 1.5 currently applied only to RA, we could identify patient subsets characterized by different CV risk profiles. In fact, patients whose SCORE remained below the cut-off regardless of the correction factor were those with the most favorable metabolic profile. On the contrary, all six patients below the cut-off who were reclassified above the cut-off using the correction factor displayed higher SUA levels. Therefore, our findings underpin the importance of assessing SUA levels in addition to all the other traditional CV risk factors in all patients with pSS and to consider applying the correction factor for CV risk assessment tools not only in RA but also in pSS. As outlined by existing recommendations on CV prevention (REF), it is acceptable to possibly overestimate CV risk in light of the benefit of better stratifying patients and identifying those requiring more aggressive intervention and thorough follow-up.

Our study displays some limitations, including its retrospective nature, the sample size and consequent small number of CV events, and the lack of subclinical atherosclerosis assessments such as carotid intima media thickness. We recognize that our results need to be validated in larger multicenter studies to ensure generalizability; however, we were the first to explore in detail the role of SUA in pSS and to demonstrate an additional facet of the complex CV scenario orchestrated by inflammation, traditional risk factors, and disease specific features.

## 5. Conclusions

In conclusion, our findings pave the way to more personalized CV preventive strategies in pSS [34] and, if their generalizability is confirmed by larger prospective studies, they will allow for the development of specific recommendations to be applied in clinical practice and ultimately reduce CV morbidity and mortality in this disease.

## Figures and Tables

**Table 1:** Characteristics of the study cohort (*N* = 105).

**Variable**	**Mean**	**SD**
Age, years	61.9	11.5
Age at diagnosis, years	56.3	12.5
Disease duration, years	5.7	5.0
ESSDAI	6.3	4.8
ClinESSDAI	6.0	4.6
ESSPRI	6.3	2.3
VAS dryness	6.0	2.4
VAS xerostomia	6.3	2.7
VAS xerophthalmia	5.8	2.9
VAS pain	5.8	3.2
VAS fatigue	7.1	2.7
Total cholesterol (mg/dL)	202.5	35.7
Triglycerides (mg/dL)	109.3	49.5
HDL-c (mg/dL)	58.0	14.4
LDL-c (mg/dL)	126.0	34.6
SUA (mg/dL)	4.4	1.1
**Variable**	***N***	**%**
Female gender	99	94
Autoantibodies		
Neither anti-Ro nor anti-La	62	59
Anti-Ro only	26	25
Both anti-Ro and anti-La	17	16
Rheumatoid factor	25	24
Never smoked	75	71
Former smoker	19	18
Current smoker	11	11
Obesity	17	16
Arterial hypertension	46	44
Hypercholesterolemia	41	39
LDL-c > 115 mg/dL	41	39
HDL-c < 40 mg/dL	18	17
Hypertriglyceridemia	12	11
Type 2 diabetes	3	3
BMI (kg/m^2^)	25.2	4.4
Obesity	17	16
Myocardial infarction	4	4
Angina	6	6
Heart failure	3	3
Stroke	3	3
TIA	14	13
PAD	5	5
CV Risk Factors		
0	31	30
1–2	48	46
>2	26	25
CV events		
0	80	76
1	17	16
>1	8	8

SD, standard deviation; BMI, body mass index; ESSDAI, EULAR Sjögren’s syndrome disease activity index; ESSPRI, EULAR Sjögren’s Syndrome Patient Reported Index; VAS, visual analog scale; TIA, transient ischemic attack; PAD, peripheral arterial disease; LDL-c, low-density lipoprotein cholesterol; HDL-c, high-density lipoprotein cholesterol; CV, cardiovascular; SUA, serum uric acid.

**Table 2:** Characteristics of the study cohort (*N* = 105) according to serum uric acid levels.

	**SUA < 4.79 mg/dL (*N* = 81)**		**SUA ≥ 4.79 mg/dL (*N* = 24)**		
**Variable**	**Mean**	**SD**	**Mean**	**SD**	***p* Value**
Age, years	62.6	12.1	59.7	9.0	0.167
Age at diagnosis, years	56.9	13.2	54.1	10.1	0.254
Disease duration, years	5.9	5.2	4.8	4.0	0.971
ESSDAI	5.5	4.2	9.0	5.6	0.005
ClinESSDAI	5.2	4.1	8.8	5.4	0.003
ESSPRI	6.3	2.3	6.5	2.3	0.662
VAS dryness	6.1	2.4	6.0	2.4	0.903
VAS xerostomia	6.3	2.6	6.2	3.1	0.984
VAS xerophthalmia	5.8	3.0	5.7	2.6	0.677
VAS vaginal dryness	5.1	3.3	6.8	2.9	0.044
VAS pain	5.7	3.3	6.2	2.9	0.663
VAS fatigue	7.1	2.7	7.3	2.9	0.628
Total cholesterol (mg/dL)	204.5	35.7	197.6	36.3	0.561
Triglycerides (mg/dL)	98.9	38.2	134.7	64.0	0.009
HDL-c (mg/dL)	59.2	15.1	54.2	11.7	0.275
LDL-c (mg/dL)	127.6	34.6	122.4	35.4	0.6
BMI (kg/m^2^)	24.6	4.3	27.2	3.9	0.009
**Variable**	***N***	**%**	***N***	**%**	***p* Value**
Female gender	80	99	19	79	<0.001
Autoantibodies					0.408
Neither anti-Ro nor anti-La	50	62	12	50	
Anti-Ro only	17	21	9	38	
Both anti-Ro and anti-La	14	17	3	13	
Rheumatoid factor	19	23	6	25	0.876
Smoking habit					0.014
Never	63	78	12	50	
Former	10	12	9	38	
Current	8	10	3	13	
Obesity	9	11	8	33	0.023
Arterial hypertension	32	40	14	58	0.159
Hypercholesterolemia	31	38	10	42	0.814
LDL-c > 115 mg/dL	31	38	10	42	0.814
HDL-c < 40 mg/dL	13	16	5	21	
Hypertriglyceridemia	6	7	6	25	0.028
Type 2 diabetes	2	2	1	4	0.545
Myocardial infarction	3	4	1	4	0.917
Stroke	0	0	3	13	0.011
TIA	11	14	3	13	0.891
Heart failure	2	2	1	4	0.661
Angina	4	5	2	8	0.618
PAD	3	4	2	8	0.321
Hydroxychloroquine	39	48	14	58	0.49
Glucocorticoids	13	16	6	25	0.37
Other immunosuppressant	20	25	4	17	
CV Risk Factors					0.024
0	27	33	4	17	
1–2	38	47	10	42	
>2	16	20	10	42	
CV events					0.07
No	65	80	15	63	
Yes	16	20	9	38	

SD, standard deviation; BMI, body mass index; ESSDAI, EULAR Sjögren’s syndrome disease activity index; ESSPRI, EULAR Sjögren’s Syndrome Patient Reported Index; VAS, visual analog scale; TIA, transient ischemic attack; PAD, peripheral arterial disease; LDL-c, low-density lipoprotein cholesterol; HDL-c, high-density lipoprotein cholesterol; CV, cardiovascular; SUA, serum uric acid.

**Table 3:** Characteristics of the study cohort (*N* = 105) according to the presence or absence of arterial hypertension (AH).

	**Without Arterial Hypertension**		**With Arterial Hypertension**		
	**Number of Patients = 59**		**Number of Patients = 46**		
**Variable**	**Mean**	**SD**	**Mean**	**SD**	***p* Value**
Age, years	59.0	13.0	65.7	8.0	0.009
Age at diagnosis, years	53.9	13.6	59.3	10.3	0.036
Disease duration, years	5.2	5.4	6.4	4.3	0.014
ESSDAI	5.8	4.4	6.9	5.3	0.317
ClinESSDAI	5.5	4.2	6.6	5.1	0.3
ESSPRI	6.0	2.2	6.6	2.3	0.147
VAS dryness	5.8	2.4	6.3	2.4	0.335
VAS xerostomia	6.0	2.8	6.6	2.5	0.301
VAS xerophthalmia	5.6	2.9	6.0	3.0	0.512
VAS vaginal dryness	5.0	3.2	5.8	3.4	0.221
VAS pain	5.6	3.2	6.1	3.2	0.479
VAS fatigue	6.9	2.7	7.5	2.8	0.137
Total cholesterol (mg/dL)	194.7	29.4	209.6	39.6	0.111
Triglycerides (mg/dL)	98.2	44.0	119.0	52.6	0.035
HDL-c (mg/dL)	62.1	15.3	53.9	12.5	0.043
LDL-c (mg/dL)	117.9	31.2	132.2	36.2	0.085
BMI (kg/m^2^)	24.4	4.4	26.1	4.3	0.020
**Variable**	***N***	**%**	***N***	**%**	***p* Value**
Female gender	56	95	43	93	0.753
Autoantibodies					0.676
Neither anti-Ro nor anti-La	37	63	25	54	
Anti-Ro only	13	22	13	28	
Both anti-Ro and anti-La	9	15	8	17	
Rheumatoid factor	13	22	12	26	0.651
Smoking habit					0.04
Never	45	76	30	65	
Former	6	10	13	28	
Current	8	14	3	7	
Obesity	8	14	9	20	0.435
Hypercholesterolemia	14	24	27	56	<0.001
LDL-c > 115 mg/dL	12	22	29	63	<0.001
HDL-c < 40 mg/dL	8	14	10	22	0.341
Hypertriglyceridemia	4	7	8	17	0.124
Type 2 diabetes	0	0	3	7	0.08
Myocardial infarction	1	2	3	6	0.317
Angina	1	2	5	11	0.08
Heart failure	1	2	2	4	0.580
Any cardiac event	2	3	8	17	0.02
Stroke	1	2	2	4	0.418
TIA	7	12	7	15	0.774
PAD	2	3	3	6	
CV Risk Factors other than hypertension					<0.0001
0	31	53	11	24	
1–2	23	39	26	56	
>2	5	8	9	20	
Previous CV event(s)	9	17	16	33	0.02

SD, standard deviation; BMI, body mass index; ESSDAI, EULAR Sjögren’s syndrome disease activity index; ESSPRI, EULAR Sjögren’s Syndrome Patient Reported Index; VAS, visual analog scale; TIA, transient ischemic attack; PAD, peripheral arterial disease; LDL-c, low-density lipoprotein cholesterol; HDL, high-density lipoprotein cholesterol; CV, cardiovascular.

**Table 4:** Characteristics of hypertensive patients (*N* = 46) according to serum uric acid levels.

	**SUA < 4.79 mg/dL (*N* = 32)**		**SUA ≥ 4.79 mg/dL (*N* = 14)**		
**Variable**	**Mean**	**SD**	**Mean**	**SD**	***p* Value**
Age, years	66.9	8.8	63.1	5.0	0.12
Age at diagnosis, years	60.5	10.8	56.6	8.9	0.19
Disease duration, years	6.5	4.5	6.1	3.9	0.97
ESSDAI	5.5	4.1	10.1	6.4	0.02
ClinESSDAI	5.2	4.0	9.8	6.1	0.02
ESSPRI	6.6	2.2	6.6	2.6	1.00
VAS dryness	6.6	2.2	5.7	2.8	0.32
VAS xerostomia	6.9	2.2	5.9	3.1	0.25
VAS xerophthalmia	6.2	2.9	5.6	3.1	0.48
VAS vaginal dryness	5.3	3.6	7.1	2.6	0.20
VAS pain	5.9	3.2	6.6	3.0	0.50
VAS fatigue	7.5	2.8	7.4	2.9	0.98
Total cholesterol (mg/dL)	213.1	41.1	202.5	37.1	0.57
Triglycerides (mg/dL)	113.5	40.7	129.1	70.4	0.65
HDL-c (mg/dL)	53.3	14.2	55.4	6.3	0.61
LDL-c (mg/dL)	135.3	37.6	126.0	34.2	0.63
BMI (kg/m^2^)	25.0	3.9	28.7	4.2	0.03
**Variable**	***N***	**%**	***N***	**%**	***p* Value**
Female gender	31	97	12	86	0.216
Autoantibodies					0.246
Neither anti-Ro nor anti-La	18	56	7	50	
Anti-Ro only	7	22	6	43	
Both anti-Ro and anti-La	7	22	1	7	
Rheumatoid factor	10	31	2	14	0.294
Smoking habit					0.02
Never	25	78	5	36	
Former	6	19	7	50	
Current	1	3	2	14	
Obesity	3	9	6	43	0.015
Hypercholesterolemia	20	62	7	50	0.522
LDL-c > 115 mg/dL	21	66	8	57	0.742
HDL-c < 40 mg/dL	8	25	2	14	
Hypertriglyceridemia	6	19	2	14	0.54
Type 2 diabetes	2	6	1	7	
Myocardial infarction	2	6	1	7	0.673
Stroke	0	0	2	14	0.088
TIA	4	12	3	21	0.658
Heart failure	1	3	1	7	0.521
Angina	3	9	2	14	0.633
PAD	2	6	1	7	0.910
CV Risk Factors other than hypertension					0.18
0	10	31	1	7	
1–2	18	56	8	57	
2–3	13	41	7	50	
>3	0	0	1	7	
CV events	8	25	7	50	0.18

SD, standard deviation; BMI, body mass index; ESSDAI, EULAR Sjögren’s syndrome disease activity index; ESSPRI, EULAR Sjögren’s Syndrome Patient Reported Index; VAS, visual analog scale; TIA, transient ischemic attack; PAD, peripheral arterial disease; LDL-c, low-density lipoprotein cholesterol; HDL-c, high-density lipoprotein cholesterol; CV, cardiovascular; SUA, serum uric acid.

**Table 5:** Characteristics of patients eligible for SCORE calculation (*N* = 79).

	**Eligible for SCORE (*N* = 79)**		**Not Eligible for SCORE (*N* = 26)**		
**Variable**	**Mean**	**SD**	**Mean**	**SD**	***p* Value**
Age, years	61.3	11.7	63.8	11.0	0.389
Age at diagnosis, years	55.8	12.7	57.7	12.1	0.506
Disease duration, years	5.8	5.2	5.5	4.3	0.946
ESSDAI	6.2	4.7	6.5	5.2	0.884
ClinESSDAI	5.9	4.6	6.2	4.9	0.875
ESSPRI	6.0	2.2	7.3	2.1	0.018
VAS dryness	5.8	2.3	6.8	2.7	0.086
VAS xerostomia	6.1	2.6	6.7	2.8	0.362
VAS xerophthalmia	5.5	2.9	6.8	2.8	0.037
VAS vaginal dryness	5.2	3.5	6.2	2.6	0.283
VAS pain	5.4	3.2	7.0	2.7	0.03
VAS fatigue	6.9	2.9	8.0	2.0	0.106
Total cholesterol (mg/dL)	194.9	31.4	228.3	38.3	0.002
Triglycerides (mg/dL)	97.7	32.6	147.6	73.5	0.005
HDL-c (mg/dL)	58.7	13.8	55.5	16.7	0.709
LDL-c (mg/dL)	119.6	30.7	150.8	38.9	0.014
SUA (mg/dL)	4.3	1.0	4.9	1.2	0.05
BMI (kg/m^2^)	24.5	4.0	27.5	4.9	0.005
**Variable**	***N***	**%**	***N***	**%**	***p* Value**
Female gender	76	96	23.0	88	0.160
Autoantibodies					0.234
Neither anti-Ro nor anti-La	47	60	15	58	
Anti-Ro only	17	21	9	34	
Both anti-Ro and anti-La	15	19	2	8	
Rheumatoid factor	19	24	6	23	0.574
Smoking habit					0.42
Never	59	75	16	62	
Former	13	16	6	23	
Current	7	9	4	15	
Obesity	9	11	8	31	0.031
Hypertension	30	38	16	62	0.04
Hypercholesterolemia	23	29	18	69	<0.001
LDL-c > 115 mg/dL	28	35			0.241
HDL-c < 40 mg/dL	9	11	9	35	
Hypertriglyceridemia	5	6	7	27	0.009
CV Risk Factors					
0	28	35	3	11	0.0001
1–2	38	48	10	38	
2–3	26	33	12	46	
>3	2	3	8	31	

SD, standard deviation; BMI, body mass index; ESSDAI, EULAR Sjögren’s syndrome disease activity index; ESSPRI, EULAR Sjögren’s Syndrome Patient Reported Index; VAS, visual analog scale; LDL-c, low-density lipoprotein cholesterol; HDL-c, high-density lipoprotein cholesterol; CV, cardiovascular; SUA, serum uric acid.

## Data Availability

All relevant data are included in the main manuscript and Supplementary Materials.

## Supplementary Materials

The following supporting information can be downloaded at: https://www.mdpi.com/article/10.3390/nu15071563/s1. Table S1: Prevalence of disease manifestations according to serum uric acid level (SUA) in the full cohort (*N* = 105); Table S2: Clinical characteristics of the full cohort (*N* = 105) stratified by different serum uric acid cut-off values; Table S3: Prevalence of disease manifestations according to various cut-off values of serum uric acid level (SUA) in the full cohort (*N* = 105).
