# Supplementary material for: The Interplay between Cardiovascular Risk, Cardiovascular Events, and Disease Activity in Primary Sjögren’s Syndrome: Is Uric Acid the Missing Link?
Source: Nutrients. 2023 Mar 23;15(7):1563. doi: 10.3390/nu15071563 (PMC10096655; doi:10.3390/nu15071563)
Supplement: Supplementary file 1 [file nutrients-15-01563-s001.zip › nutrients-2248545-supplementary.pdf]

Supplementary Material

Supplementary Table S1. Prevalence of disease manifestations according to serum uric acid level (SUA) in the full cohort (N=105).

	**SUA < 4.79 mg/dL (N=81)**		**SUA ≥ 4.79 mg/dL (N=24)**		
**ESSDAI DOMAINS**	**N**	**%**	**N**	**%**	**p value**
Constitutional	11	14	7	29	0.119
Lymphadenopathy	22	27	9	38	0.445
Glandular	11	14	8	33	0.037
Articular	59	73	17	71	0.847
Cutaneous	6	7	2	8	0.881
Pulmonary	0	0	5	21	<0.001
Renal	0	0	0	0	-
Muscular	0	0	0	0	-
Peripheral nervous system	13	16	5	21	0.553
Central nervous system	0	0	0	0	-
Hematological	7	9	10	42	<0.001
Biological	22	27	6	25	0.833

ESSDAI, EULAR Sjögren's syndrome disease activity index; SUA, serum uric acid.

Supplementary Table S2. Clinical characteristics of the full cohort (N=105) stratified by different serum uric acid cut-off values.

	SUA <4.79 mg/dL		4.79 mg dL ≤SUA<5.6 mg/dL		SUA ≥ 5.6 mg/dL		
	N=81		N=17		N=7		
	**Mean**	**SD**	**Mean**	**SD**	**Mean**	**SD**	**p value**
**ESSDAI**	5.5	4.2	8.7	4.6	9.9	8.0	0.019
**ClinESSDAI**	5.2	4.1	8.5	4.6	9.4	7.3	0.013
**ESSPRI**	6.3	2.3	6.6	2.0	6.1	3.3	0.882
**VAS dryness**	6.1	2.4	6.2	2.4	5.3	2.7	0.648
**VAS xerostomia**	6.3	2.6	6.3	3.4	5.8	2.4	0.699
**VAS xerophtalmia**	5.8	3.0	6.0	2.4	4.8	3.3	0.670
**VAS vaginal dryness**	5.1	3.3	6.6	3.2	7.2	1.9	0.124
**VAS pain**	5.7	3.3	6.1	2.5	6.3	4.1	0.794
**VAS fatigue**	7.1	2.7	7.6	2.6	6.5	3.7	0.757

SD, standard deviation; ESSDAI, EULAR Sjögren's syndrome disease activity index; ESSPRI, EULAR Sjögren's Syndrome Patient Reported Index; VAS, visual analog scale; SUA, serum uric acid.

Supplementary Table S3. Prevalence of disease manifestations according to various cut-off values of serum uric acid level (SUA) in the full cohort (N=105).

	**SUA <4.79 mg/dL**	**4.79 mg dL ≤SUA<5.6 mg/dL**	**SUA ≥ 5.6 mg/dL**	
	**N=81**	**N=17**	**N=7**	
**ESSDAI DOMAINS**	**N (%)**			**p value**
Constitutional	11 (13)	4 (23)	3 (43)	0.11
Lymphadenopathy	22 (27)	6 (35)	3 (43)	0.58
Glandular	11 (13)	4 (23)	4 (57)	0.01
Articular	59 (73)	12 (70)	5 (71)	0.98
Cutaneous	6 (7)	1 (6)	1 (14)	0.77
Pulmonary	0 (0)	3 (18)	2 (28)	<0.001
Renal	0 (0)	0 (0)	0 (0)	-
Muscular	0 (0)	0 (0)	0 (0)	-
Peripheral Nervous system	13 (16)	4 (23)	1 (14)	0.74
Central nervous system	0 (0)	0 (0)	0 (0)	-
Hematological	7 (9)	8 (47)	2 (28)	<0.001
Biological	22 (27)	4 (23)	2 (28)	0.95

ESSDAI, EULAR Sjögren's syndrome disease activity index; SUA, serum uric acid.
